# Supplementary material for: Spatio-temporal evolution of acoustic emission events and initiation of stress fields in the fracturing of rock mass around a roadway under cyclic high-stress loading
Source: PLoS One. 2023 Sep 28;18(9):e0286005. doi: 10.1371/journal.pone.0286005 (PMC10538668; doi:10.1371/journal.pone.0286005)
Supplement: Attachment — Submitted filename: Comments.docx [file pone.0286005.s001.docx]

Comments to the Author

This manuscript investigated the non-linear flow in sandstone with cracks through comprehensive experiments. Understanding fluid flow in natural porous rock is a key issue for many fields, including shale gas fracking, wastewater geological disposal, slope stability, etc. Numerous natural fractures widely exist in rock projects. Through experiments of many specimens with pre-cracks, the authors found the Forchheimer coefficient is always negative for sandstones with cracks. Reports in the negative Forchheimer coefficient are limited. The findings of this study are interesting and meaningful. But there are still some issues that need to be explained. Manuscripts will be accepted after revision. Some comments are as follows:

(1) About the Section 4 - Analysis of test results, it seems more appropriate to break it down into results and discussions; Figure 8 need to be redesigned and drawn, there are too many elements in the figures, and the improperly placed legends lead to confusion with data points.

(2) Line 287-290: the angle of the sample with a single fracture has a greater impact on the seepage characteristic of the pore-fracture media, while the angles of the sandstone samples with T-shaped and Y-shaped fractures have a relatively small impact. This is an interesting finding. Can you briefly explain it? It is perhaps because the angle of a single fracture dominates the flow.

(3) English needs heavy editing in order to make the paper suitable for the readers.
