# Supplementary material for: Spatio-temporal evolution of acoustic emission events and initiation of stress fields in the fracturing of rock mass around a roadway under cyclic high-stress loading
Source: PLoS One. 2023 Sep 28;18(9):e0286005. doi: 10.1371/journal.pone.0286005 (PMC10538668; doi:10.1371/journal.pone.0286005)
Supplement: Attachment — Submitted filename: Response to Reviewers.docx [file pone.0286005.s002.docx]

Reviewer #1

Comments to the Author

This manuscript investigated the non-linear flow in sandstone with cracks through comprehensive experiments. Understanding fluid flow in natural porous rock is a key issue for many fields, including shale gas fracking, wastewater geological disposal, slope stability, etc. Numerous natural fractures widely exist in rock projects. Through experiments of many specimens with pre-cracks, the authors found the Forchheimer coefficient is always negative for sandstones with cracks. Reports in the negative Forchheimer coefficient are limited. The findings of this study are interesting and meaningful. But there are still some issues that need to be explained. Manuscripts will be accepted after revision. Some comments are as follows:

(1) About the Section 4 - Analysis of test results, it seems more appropriate to break it down into results and discussions; Figure 8 need to be redesigned and drawn, there are too many elements in the figures, and the improperly placed legends lead to confusion with data points.

**Response:** Thanks for the review by reviewer. However, in this manuscript, the serial numbers of the pictures are from Figures 1 to 7, and there is no Figure 8, so please further explain which picture is mentioned.

(2) Line 287-290: the angle of the sample with a single fracture has a greater impact on the seepage characteristic of the pore-fracture media, while the angles of the sandstone samples with T-shaped and Y-shaped fractures have a relatively small impact. This is an interesting finding. Can you briefly explain it? It is perhaps because the angle of a single fracture dominates the flow.

**Response:** Thanks for the review by reviewer. In the unmodified manuscript, lines 287 to 290 are as follows：

Therefore, please further explain what this question wants to express.

(3) English needs heavy editing in order to make the paper suitable for the readers.

**Response:** Thanks for the review by reviewer. The full text has been checked again, some wrong statements have been modified, and native speakers have been hired for language review.

Reviewer #2

The authors carried out a series of loading and unloading experiments of roadway rock mass with high precision acoustic emission monitoring technology to study the fracture mechanism of roadway rock mass under cyclic load and the breeding process of internal stress field under high stress environment. The study provides reference for roadway support and disaster prevention in deep mine, and is suitable for publication in Plos One journal. Some suggestions are given below:

(1)The abstract can be simplified.

**Response:** Thanks for the review by reviewer, As the Reviewer has suggested, the abstract has been modified. Revised as follows:

To study fracture mechanisms and initiation of stress fields in the rock mass around a roadway subjected to cyclic stress, a series of loading and unloading tests were conducted on the rock mass around the roadway by using high-precision acoustic emission (AE) monitoring. The results show that intense AE activities occur in a specimen during cyclic load-holding at different levels. With the increase in the number of cycles, the overall stability of the specimen gradually decreases. In the cyclic loading and unloading process, the specimen exhibits a Kaiser effect. As the number of cycles increases, more AE events occur in the unloading stage and a Felicity effect is manifest. The spatial distribution of AE events is related to the stress regime and structure of the specimen, crack propagation in the roadway exhibits directionality due to effects of the principal stress. High stress is conducive to microcrack initiation and propagation in the specimen, which accelerates damage accumulation and macrofracture formation in a rock mass. The research provides a reference for roadway support work and disaster prevention and control in deep mines.

(2)It is suggested to conduct a novelty search on the references in the paper. Please add relevant research in relevant fields in recent years.

**Response:** Thanks for the review by reviewer, References have been supplemented and updated.

1. Manchao He, Qi Wang, Qunying Wu. Innovation and future of mining rock mechanics. Journal of Rock Mechanics and Geotechnical Engineering. 2021, 13(1): 1-21. doi: https://doi.org/10.1016/j.jrmge.2020.11.005
2. Dawei Wu, Long An, Yuanhui Li, et al. Evolution models of the damage to the near-field rocks surrounding the drift under the disturbance caused by extraction of the longhole stope. Engineering Failure Analysis. 2022, 139. doi: https://doi.org/10.1016/j.engfailanal.2022.106500
3. Yingtao Si, Jianpo Liu, Fengtian Li, et al. Experimental and theoretical investigation of roadway fracture induced by static-blast loading using acoustic emission. Engineering Failure Analysis. 2023, 143. doi: https://doi.org/10.1016/j.engfailanal.2022.106909
4. Beibei Yang , Mingming He, Zhiqiang Zhang, et al. Experimental investigation and empirical model on damping properties of rock under multistage cyclic loading. Soil Dynamics and Earthquake Engineering. 2022, 163. doi: <https://doi.org/10.1016/j.soildyn.2022.107557>
5. Shucai Li, Jie Hu, Florian Amann, et al. A multifunctional rock testing system for rock failure analysis under different stress states: Development and application. Journal of Rock Mechanics and Geotechnical Engineering. 2022, 14(5): 1531-1544. doi: <https://doi.org/10.1016/j.jrmge.2021.12.017>
6. Yulong Jiang, Weiguo Liang , Tingting Cai , et al. Fracture growth and acoustic emission response in natural coal-rock blocks with different stress, fracturing medium and injection rates. Journal of Petroleum Science and Engineering. 2023, 220: 1531-1544. doi: <https://doi.org/10.1016/j.petrol.2022.111228>
7. Yuelin Xia, Xiating Feng , Chengxiang Yang , et al. Mechanism of excavation-induced cracking of the protective layer of a rock bench in a large underground powerhouse under high tectonic stress. Engineering Geology. 2023, 312. doi: <https://doi.org/10.1016/j.enggeo.2022.106951>
8. Xiating Feng, Xiaojun Yu, Yangyi Zhou, et al. A rigid true triaxial apparatus for analyses of deformation and failure features of deep weak rock under excavation stress paths. Journal of Rock Mechanics and Geotechnical Engineering. 2022. doi: <https://doi.org/10.1016/j.jrmge.2022.08.009>
9. Hao Lin , Jianfeng Liu , Jianxiong Yang, et al. Analysis of damage characteristics and energy evolution of salt rock under triaxial cyclic loading and unloading. Journal of Energy Storage. 2023, 56. doi: <https://doi.org/10.1016/j.est.2022.106145>
10. H. Ren, Y. Zhu, P. Wang, et al. Experimental study on mechanical characteristics of unloaded damaged white sandstone before peak. Arab. J. Geosci. 2020, 13(17). doi: <https://doi.org/10.1007/s12517-020-05856-z>
11. Vaneghi R G, Ferdosi B, Okoth A D, et al. Strength degradation of sandstone and granodiorite under uniaxial cyclic loading. Journal of Rock Mechanics and Geotechnical Engineering. 2018, 10(1): 117-126. doi: <https://doi.org/10.1016/j.jrmge.2017.09.005>
12. Chonghong Ren , Jin Yu , Xueying Liu, et al. Cyclic constitutive equations of rock with coupled damage induced by compaction and cracking. International Journal of Mining Science and Technology. 2022, 32(5): 1153-1165. doi: <https://doi.org/10.1016/j.ijmst.2022.06.010>
13. Q.B. Lin, P. Cao, S.Y. Mao, et al. Fatigue behaviour and constitutive model of yellow sandstone containing pre-existing surface crack under the uniaxial cyclic loading. Theor Appl Fract Mech. 2020, 109. doi: <https://doi.org/10.1016/j.tafmec.2020.102776>
14. Shengqi Yang , Pengfei Yin , Yuanchao Zhang, et al. Failure behavior and crack evolution mechanism of a non-persistent jointed rock mass containing a circular hole. International Journal of Rock Mechanics and Mining Sciences. 2019, 114: 101-121. doi: <https://doi.org/10.1016/j.ijrmms.2018.12.017>
15. Shibing Huang , Yanzhang Liu , Yunlin Guo, et al. Strength and failure characteristics of rock-like material containing single crack under freeze-thaw and uniaxial compression. Cold Regions Science and Technology. 2019, 132: 1-10. doi: <https://doi.org/10.1016/j.coldregions.2019.03.013>
16. Zhiqiang Zhou, Yu Zhao , Jing Bi, et al. Shear mechanical properties and failure modes of rock with V-shaped intersecting double-cracks. Theoretical and Applied Fracture Mechanics. 2023, 124 doi: <https://doi.org/10.1016/j.tafmec.2023.103755>
17. Qiquan Xiong , Qing Lin , Yue Gao, et al. Fundamental physics distinguishes the initial stage acoustic emission (AE) behavior between compressive and fracture toughness tests in rock. Engineering Fracture Mechanics. 2022, 275 doi: <https://doi.org/10.1016/j.engfracmech.2022.108829>
18. Kun Du, Xuefeng Li , Ming Tao, et al. Experimental study on acoustic emission (AE) characteristics and crack classification during rock fracture in several basic lab tests. International Journal of Rock Mechanics and Mining Sciences. . 2020, 133 doi: <https://doi.org/10.1016/j.ijrmms.2020.104411>
19. Jianpo L, Yuanhui L, Shida X, et al. Cracking mechanisms in granite rocks subjected to uniaxial compression by moment tensor analysis of acoustic emission. Theoretical and Applied Fracture Mechanics. 2015, 75: 151-159. doi: https://doi.org/10.1016/j.tafmec.2014.12.006
20. Zhang Z, Wang E, Li N, et al. Damage evolution analysis of coal specimens under cyclic loading based on single-link cluster method. Journal of Applied Geophysics. 2018, 152: 56-64. doi: <https://doi.org/10.1016/j.jappgeo.2018.03.014>
21. Liu J P , Zhang C Y , Si Y T , et al. Temporal-spatial evolution of acoustic emission during progressive fracture processes around tunnel triggered by blast-induced disturbances under uniaxial and biaxial compression. Tunnelling and Underground Space Technology, 2020, 96. doi: <https://doi.org/10.1016/j.tust.2019.103229>
22. Long Y , Liu J P , Lei G , et al. Progressive fracture processes around tunnel triggered by blast disturbances under biaxial compression with different lateral pressure coefficients. Transactions of Nonferrous Metals Society of China. 2020, 30(9):2518-2535. doi: <https://doi.org/10.1016/S1003-6326(20)65398-5>
23. Jianpo L, Shida X, Yuanhui L. Analysis of rock mass stability according to power-law attenuation characteristics of acoustic emission and microseismic activities. Tunnelling and Underground Space Technology. 2019, 83: 303-312. doi: <https://doi.org/10.1016/j.tust.2018.09.023>
24. Linqi Huang, Xin Wu, Xibing Li, et al. Influence of sensor array on MS/AE source location accuracy in rock mass. Transactions of Nonferrous Metals Society of China. 2023, 33(1): 254-274. doi: https://doi.org/10.1016/S1003-6326(22)66104-1
25. Yuanhui Li, Gang Lei, Shida Xv, et al. The spatial-temporal evolution law of microseismic activities in the failure process of deep rock masses. Journal of Applied Geophysics. 2018, 154: 1-10. doi: [https://doi.org/10.1016/j.jappgeo.2018.04.024](https://webvpn.neu.edu.cn/https/77726476706e69737468656265737421f4f848d228226f/10.1016/j.jappgeo.2018.04.024)
26. Xuezhong Chen, Yane Li, Lijuan Chen. Investigation on variations of apparent stress in the region in and around the rupture volume preceding the occurrence of the 2021 Alaska M_W_8.2 earthquake. Earthquake Science. 2022, 35(3): 147-160. doi: https://doi.org/10.1016/j.eqs.2022.06.002
27. Yane Li, Xuezhong Chen, Lijuan Chen. Joint analysis of b-value and apparent stress before the 2011 M_W_9.0 Tohoku-Oki, Japan earthquake. 2021. 34(4): 323-333. doi: https://doi.org/10.29382/eqs-2021-0036
28. Brown Laura, Hudyma Martin. Identification of Stress Change Within a Rock Mass Through Apparent Stress of Local Seismic Events. Rock Mechanics and Rock Engineering. 2017, 50(1): 81-88. doi: [https://doi.org/](https://webvpn.neu.edu.cn/https/77726476706e69737468656265737421f4f848d228226f/10.1016/j.tecto.2006.08.013)10.1007/s00603-016-1092-z
29. Brune J N. Tectonic stress and the spectra of seismic shear waves from earthquakes. Journal of geophysical research. 1970, 75(26): 4997-5009. doi: [https://doi.org/](https://webvpn.neu.edu.cn/https/77726476706e69737468656265737421f4f848d228226f/10.1016/j.tecto.2006.08.013)
    10.1029/JB075i026p04997
30. Archuleta R J, Cranswick E, Mueller C, et al. Source parameters of the 1980 mammoth lakes, california, earthquake sequence. Journal of Geophysical Research: Solid Earth. 1982, 87 (B6): 4595-4607.

(3)It is well known that engineering rock mass has heterogeneous characteristics, and its strength characteristics are also the main factors affecting the spatio-temporal evolution of microcracking or on the initiation, expansion, and transfer characteristics of stress fields in rock. In this paper, M425 cement is used to simulate the engineering rock mass, and the engineering rock mass is regarded as isotropic material with good homogeneity. Please explain whether the test results are representative and whether the conclusions obtained are affected by the cement model.

**Response:** Thanks for the review by reviewer. The failure of a rock after being loaded is essentially a process in which weak surfaces such as microcracks, cracks, and defects gradually aggregate and connect within the rock until they develop into large-scale failure. The mechanism of its formation is similar to the fracture caused by indoor cement mortar test blocks under load, and can be seen as a phenomenon of rock failure. Cement mortar has good uniformity and isotropy, and its strength can be adjusted according to the capacity of the loading equipment. It is often used as a material to simulate engineering rock masses. As per literature:

31. Liu J P , Zhang C Y , Si Y T , et al. Temporal-spatial evolution of acoustic emission during progressive fracture processes around tunnel triggered by blast-induced disturbances under uniaxial and biaxial compression. Tunnelling and Underground Space Technology, 2020, 96. doi: <https://doi.org/10.1016/j.tust.2019.103229>
32. Long Y , Liu J P , Lei G , et al. Progressive fracture processes around tunnel triggered by blast disturbances under biaxial compression with different lateral pressure coefficients. Transactions of Nonferrous Metals Society of China. 2020, 30(9):2518-2535. doi: <https://doi.org/10.1016/S1003-6326(20)65398-5>

This physical experiment is a study on the regularity of tunnel failure. The conclusion obtained by using cement mortar as a model material instead of rock has a certain representativeness, and its regularity conclusion will not be affected.

4. Modify the image format to make the picture clearer.

**Response:**  Thanks for the review by reviewer. Through careful review of all the pictures in the article, we made modifications as suggested.

5. It is necessary to appropriately increase the quantified conclusion.

**Response:**  Thanks for the review by reviewer. We add the quantified conclusion as follows:

Both stress loading and stress holding (or unloading) will cause damage and destruction in the specimen. According to statistics, for S1-1 sample, the proportion of AE signals generated by stress loading and stress holding is 91.32% and 8.68% respectively. For S2-3 sample, the proportion of AE signals generated by stress loading and stress holding is 86.21% and 13.79% respectively. For S3-2 sample, the proportion of AE signals generated by stress loading and stress unloading is 92.25% and 7.75% respectively. For S4-1 specimen, the proportion of AE signals generated by stress loading and stress unloading is 87.52% and 12.48% respectively. The test results of different groups of samples show that the higher the stress level of rock mass is, the higher the AE signal ratio generated by stress holding (or unloading) is, and the stress loading is more likely to damage and destroy the rock mass in high stress state.
