# Supplementary material for: Spatio-temporal evolution of acoustic emission events and initiation of stress fields in the fracturing of rock mass around a roadway under cyclic high-stress loading
Source: PLoS One. 2023 Sep 28;18(9):e0286005. doi: 10.1371/journal.pone.0286005 (PMC10538668; doi:10.1371/journal.pone.0286005)
Supplement: Attachment — Submitted filename: PONE-D-22-35616.docx [file pone.0286005.s003.docx]

The authors carried out a series of loading and unloading experiments of roadway rock mass with high precision acoustic emission monitoring technology to study the fracture mechanism of roadway rock mass under cyclic load and the breeding process of internal stress field under high stress environment. The study provides reference for roadway support and disaster prevention in deep mine, and is suitable for publication in Plos One journal.
